# Cellular senescence and disrupted proteostasis induced by myotube atrophy are prevented with low-dose metformin and leucine cocktail

**DOI:** 10.18632/aging.204600

**Published:** 2023-03-20

**Authors:** Jonathan J. Petrocelli, Naomi M.M.P. de Hart, Marisa J. Lang, Elena M. Yee, Patrick J. Ferrara, Dennis K. Fix, Amandine Chaix, Katsuhiko Funai, Micah J. Drummond

**Affiliations:** 1 Department of Physical Therapy and Athletic, University of Utah, Salt Lake City, UT 84112, USA; 2 Department of Nutrition and Integrative Physiology, University of Utah, Salt Lake City, UT 84112, USA; 3 Molecular Medicine Program, University of Utah, Salt Lake City, UT 84112, USA

**Keywords:** skeletal muscle atrophy, inflammation, senolytic, AMPK, protein breakdown

## Abstract

Aging coincides with the accumulation of senescent cells within skeletal muscle that produce inflammatory products, known as the senescence-associated secretory phenotype, but the relationship of senescent cells to muscle atrophy is unclear. Previously, we found that a metformin + leucine (MET+LEU) treatment had synergistic effects in aged mice to improve skeletal muscle structure and function during disuse atrophy. Therefore, the study’s purpose was to determine the mechanisms by which MET+LEU exhibits muscle atrophy protection *in vitro* and if this occurs through cellular senescence. C2C12 myoblasts differentiated into myotubes were used to determine MET+LEU mechanisms during atrophy. Additionally, aged mouse single myofibers and older human donor primary myoblasts were individually isolated to determine the translational potential of MET+LEU on muscle cells. MET+LEU (25 + 125 μM) treatment increased myotube differentiation and prevented myotube atrophy. Low concentration (0.1 + 0.5 μM) MET+LEU had unique effects to prevent muscle atrophy and increase transcripts related to protein synthesis and decrease transcripts related to protein breakdown. Myotube atrophy resulted in dysregulated proteostasis that was reversed with MET+LEU and individually with proteasome inhibition (MG-132). Inflammatory and cellular senescence transcriptional pathways and respective transcripts were increased following myotube atrophy yet reversed with MET+LEU treatment. Dasatinib + quercetin (D+Q) senolytic prevented myotube atrophy similar to MET+LEU. Finally, MET+LEU prevented loss in myotube size in alternate *in vitro* models of muscle atrophy as well as in aged myofibers while, in human primary myotubes, MET+LEU prevented reductions in myonuclei fusion. These data support that MET+LEU has skeletal muscle cell-autonomous properties to prevent atrophy by reversing senescence and improving proteostasis.

## INTRODUCTION

In aged populations, skeletal muscle atrophy contributes to loss of independence [1], and all-cause mortality [2]. Muscle atrophy in aging is further compounded by low levels of physical activity and physical disuse events (hospitalizations, illness, injury) [3]. Thus, the advancement in pharmacological and nutritional approaches to prevent muscle atrophy in aging is warranted. Recently, we observed that the combination of the type 2 diabetes treatment, metformin, with the branched-chain essential amino acid, leucine, was able to protect against diminished muscle function, increased inflammation, and accumulation of fibrosis during disuse atrophy in aged mice [4]. While promising, it is currently not known if this cocktail specifically targets skeletal muscle cells and the mechanism of this possible interaction during an atrophy stimulus.

The mechanisms of skeletal muscle atrophy are complex and are dependent on the type and duration of the atrophy-causing stimulus. Nonetheless, there are shared mechanisms amongst atrophy pathologies that drive disrupted proteostasis (reduced protein synthesis and/or increased protein degradation) including increased inflammatory signaling, mitochondrial dysfunction, endoplasmic reticulum (ER) stress, autophagy, apoptosis signaling, proteasome activity, altered mechanistic target of rapamycin complex 1 (mTORC1) and 5’ AMP-activated protein kinase alpha (AMPKα) related signaling [5]. Metformin and leucine have demonstrated an ability to target many of these pathways implicated in disrupted proteostasis and thus, muscle atrophy. For instance, in skeletal muscle, metformin was able to reduce ubiquitin-proteasome markers during early hindlimb unloading [6], while leucine is a known activator of mTORC1 and protein synthesis [7]. Moreover, in skeletal muscle of aged mice during hindlimb unloading, we observed MET+LEU improved muscle function, ECM remodeling, and transcriptional pathways related to inflammation, mitochondrial dysfunction, apoptosis, and myogenesis [4].

Senescent cells accumulate with advanced age and during disease states, and their senescence-associated secretory phenotype (SASP) contributes to inflammation, ECM remodeling, mitochondrial dysfunction, and impaired muscle function [8, 9]. The connection between senescent cells and muscle atrophy is less understood. The SASP is a collection of secreted factors important in regulating skeletal muscle remodeling such as cytokines, chemokines, ECM remodeling proteins, and growth factors [9]. Cellular senescence in skeletal muscle has been characterized in resident cells (fibroblasts and macrophages) and, more recently, within myonuclei of mature muscle fibers [10]. Indeed, whole-body overexpression of the senescent marker, p21, in mice reduced muscle function and induced myofiber atrophy [8] suggesting a link between cellular senescence and muscle atrophy. Interestingly, metformin has been shown to protect against cellular senescence and the SASP in many different cell types [11–17], but is unexplored in mature skeletal muscle cells in conditions of muscle atrophy and when combined with leucine.

The purpose of this study was to identify the skeletal muscle cell-intrinsic effects of MET+LEU during an atrophy stimulus. Secondarily, we sought to determine the possible mechanisms underlying MET+LEU action on skeletal muscle cells with an emphasis on cellular senescence. We hypothesized that MET+LEU would prevent myotube atrophy through regulation of proteostasis and that MET+LEU would decrease markers of cellular senescence.

## METHODS

### Cell culture and treatments

C2C12 myoblasts (ATCC cat# CRL-1772) were grown in high glucose DMEM (Gibco cat# 11965-092) with 10% fetal bovine serum in a 37°C, 5% CO_2_ incubator. Once reaching 95–100% confluence, myoblasts were differentiated via low glucose DMEM (Gibco cat# 11885-084) with 2% horse serum for 4 days. To determine treatment effects during differentiation, metformin (MET) (Millipore Sigma, cat# 317240), leucine (LEU) (Sigma Aldrich, cat# L8000), or metformin + leucine (MET+LEU) were added at the start of differentiation. Media with and without treatments were changed on day 2 of differentiation. To determine treatment effects during myotube atrophy, following 4 days of differentiation, myotubes were deprived of 2% horse serum (serum-deprivation; SD) for 4 days and simultaneously treated with MET, LEU, MET+LEU, sildenafil (Millipore Sigma, cat# 1612561), or metformin + leucine + sildenafil (MET+LEU+SILD) while control cells remained with 2% horse serum (2% HS). For acute effects of treatments during SD, after 4 days of differentiation when myotubes were switched to serum-deprived media, cells were collected at times 15 min to 8 hr post-treatment. To determine proteasome contribution to myotube atrophy during SD, the proteasomal inhibitor MG-132 (Abcam, cat# ab147047) was added at the start of SD and introduced again with media change on day 2. To determine the effect of senolytic treatment on myotube atrophy during SD, 62.5 nM dasatinib + 12.5 μM quercetin (D+Q) (Millipore Sigma, cat# 551600 and Sigma Aldrich, cat# SML2589) were added at the start of SD and introduced again with media change on day 2. We also tested the effects of M+L on alternate models of myotube atrophy. To induce inflammatory atrophy, 100 ng/mL TNF-α (Peprotech, cat# 315-01A) was added on day 2 of differentiation and introduced again with media change on day 4; myotubes were collected on day 5 of differentiation. To induce fatty acid-induced atrophy, palmitic acid (Sigma, cat# P5585) was dissolved in ethanol and conjugated to fatty acid-free BSA (Sigma, cat# A7030) at 0.75 mM. Palmitate was added on day 4 of differentiation for 24 h.

### Myotube area and myogenic index

Myotubes were washed 3 times with 1× PBS then fixed in 4% paraformaldehyde for 30 minutes. After fixation, myotubes were washed then permeabilized in 0.25% Triton-X100 in 1× PBS for 15 minutes. Triton-x100 was washed off and myotubes were blocked in 10% horse serum (HS) in 1× PBS for 30 minutes, followed by 3 washes in 1% HS in 1× PBS. Next, myotubes were incubated in 1:100 MF20 primary antibody (Invitrogen, cat# 50-6503-82) in 1% HS for 1 hour, washed in 1% HS, then incubated in 1:10,000 DAPI for 5 minutes. 7 × 7 fields were captured at 10X objective with a fully automated wide-field light microscope (Nikon Ti, Tokyo, Japan) utilizing a high-sensitivity Andor Clara CCD camera (Belfast, UK).

Myotube area was determined utilizing ImageJ software using a size threshold of >5000 μm^2^ to obtain the percent area of myotubes. Myogenic Index was determined utilizing ImageJ software and quantifying the number of myonuclei within myotubes divided by total nuclei as previously described [18].

### Transcriptomic analysis

Total RNA was isolated by homogenizing myotubes in Qiazol Lysis Reagent (Qiagen, cat# 79306). The RNA was separated and precipitated using chloroform and isopropanol. Extracted RNA was washed with ethanol then suspended in nuclease-free water. RNA concentration was determined using an EPOCH (Take3, BioTek) spectrophotometer. Libraries were prepared with Illumina Stranded Total RNA Library Prep Ribo-Zero Plus and RNA was sequenced using Illumina NovaSeq S4 Reagent Kit v1.5 150 × 150 bp Sequencing (100 M read-pairs). Differentially expressed genes were identified using a 5% false discovery rate with DESeq2 version 1.30.00 and the hciR package. Data can be found on the Gene Expression Omnibus (GSE221941) Hallmark, KEGG, and REACTOME pathways were identified using the fast gene set enrichment analysis in MSigDB using a 10% FDR. Metascape.org was utilized to determine TRRUST enrichment analysis and protein-protein interaction maps.

### Proteasome activity assay

Proteasome activity assay was performed following the manufacturer’s instructions (Abcam, cat# ab107921) and was measured on a Varioskan Lux (Thermo Scientific) fluorescent plate reader. Briefly, 10 ug of protein from myotubes was added to a black 96-well fluorescent compatible plate with and without MG-132 proteasome inhibition for each sample. Reads with excitation of 350 nm and emission of 440 nm were performed every 2 minutes for 60 minutes. Proteasome activity rate was determined by dividing the amount of fluorescent AMC (by-product of proteasome activity) by the difference in the 60 and 30-minute timepoints, multiplied by the volume of sample added into each reaction well.

### Immunoblotting

Proteins were isolated by homogenizing myotubes in ice-cold lysis buffer (50 mM Tris-HCl pH 7.5, 150 mM NaCl, 5 mM EDTA, 1% Triton X-100, 0.1% sodium deoxycholate, 0.1% SDS, 1X protease and phosphatase inhibitor, (ThermoFisher)). Lysates were centrifuged (12,000 g for 15 min at 4°C), and supernatant was collected. Protein concentrations were determined using a BCA assay (ThermoScientific). Proteins were run through polyacrylamide gel electrophoresis and transferred onto polyvinylidene difluoride membranes or nitrocellulose membranes. Ponceau S (VWR, cat# K793) staining was conducted to confirm adequate protein transfer. Membranes were blocked 1 h in 5% BSA-TSBT at room temperature, primary antibodies were used 1:1000 in 5% BSA-TBST overnight at 4°C, and secondary antibodies (Cell Signaling Technologies, anti-mouse, cat# 7076 and anti-rabbit, cat# 7074) were mixed at 1:4000 in 5% BSA-TBST for 1 h at room temperature. Membranes were incubated with ECL Prime Western Blotting Detection Reagent (GE Healthcare, cat# RPN2236) and imaged using a ChemiDoc Imaging System (Bio-Rad) and quantified with Image Lab Software (Bio-Rad). The following primary antibodies were purchased from Cell Signaling Technologies: AMPK, cat# 2532; p-AMPK thr172, cat# 2531; ACC, cat# 3676; p-ACC ser79, cat# 11818; mTOR, cat# 2983; p-mTOR ser2448, cat# 2971; rpS6, cat# 2217; p-rpS6 ser240/244, cat# 2215; p70S6K, cat# 9202; p-p70S6K thr389, cat# 9205; GAPDH, cat# 2118; SOD2, cat# 13141; p-ULK1 ser757, cat# 6888; ULK, cat# 4776; LC3B, cat# 2775; p62, cat# 39749; Caspase3, cat# 14220; PARP, cat# 9532; PERK, cat# 5683; p-eIF2α ser51, cat# 3398; eIF2α, cat# 9722; BiP, cat# 3177; CHOP, cat# 2895; Ero1-Lα, cat# 3264; IER1α, cat# 3294. PGC-1α (cat# ST1202) and puromycin (cat# MABE343) antibodies were purchased from EMD Millipore. 4-Hydroxynonenal (4-HNE) (cat#, ab48506) and mitochondria complexes antibodies (cat#, ab110413) were purchased from Abcam.

### Mitochondrial isolation

Myotubes were lysed in ice-cold mitochondrial isolation medium (MIM) containing 300 mM sucrose, 10 mM HEPES, 1 mM EGTA, and 1 mg/ml bovine serum albumin (BSA) at pH 7.4 and gently homogenized with a Teflon pestle. Samples were then centrifuged at 800 g for 10 min at 4°C. The supernatants were transferred to fresh tubes and centrifuged again at 1,300 g for 10 min at 4°C. To achieve the mitochondrial fraction, the supernatants were transferred to new tubes and centrifuged at 12,000 g for 10 min at 4°C. The final mitochondrial pellets were resuspended in MIM buffer without BSA for experimental use.

### Mitochondrial respirometry

Isolated mitochondria were suspended in buffer Z (MES potassium salt; 105 mM, KCl 30 mM, KH2PO4 10 mM, MgCl2 5 mM, and BSA 0.5 mg/ml), and analyzed by high-resolution respirometry (Oroboros O2k Oxygraphs). Mitochondrial O_2_ consumption was measured using Oroboros oxygraphs as previously described [19].

Mitochondrial H_2_O_2_ production was measured using a Horiba Fluoromax-4/The Amplex UltraRed (10 μM)/horseradish peroxidase (3 U/ml) detection system (excitation/emission, 565:600, HORIBA Jobin Yvon Fluorolog) at 37°C. 12.5 μg of mitochondrial protein was placed into a glass cuvette with Amplex UltraRed reagents and buffer Z (with 1 mM EGTA and 23 U superoxide dismutase). A 5-min background rate was obtained before adding 10 mM succinate to the cuvette to induce H_2_O_2_ production. After 4 min, 100 μM 1,3-bis(2-chloroethyl)-1-nitrosourea (BCNU) was added to the cuvette, an inhibitor of the antioxidant glutathione reductase. After an additional 4 min, the assay was halted and the appearance of the fluorescent product was measured with excitation/emission at 565/600 nm. Mitochondrial JH_2_O_2_/JO_2_ was determined by dividing H_2_O_2_ production by O_2_ consumption using the same conditions for H_2_O_2_ production.

ATP production was measured using a FluoroMax-4 (Horiba Scientific). ATP production was coupled enzymatically to reduced nicotinamide adenine dinucleotide phosphate (NADPH). Briefly, ATP synthesis was stimulated using 0.5 mM malate, 5 mM pyruvate, 5 mM glutamate, and 5 mM succinate in the presence of 20 μM, 200 μM, or 2000 μM adenosine diphosphate (ADP). NADPH fluorescence was measured every 2 s (excitation 340 and emission 460). The ATP/O ratio was determined by dividing ATP production by O_2_ consumption using the same conditions for ATP production.

### Oxygen consumption rate and extracellular acidification rate

Mitochondrial respiration dynamics were measured via oxygen consumption rate (OCR) on a Seahorse XFe96 Analyzer (Agilent). Basal respiration was established followed by administration of 4 μM oligomycin to inhibit ATP synthase. Next, 4 μM FCCP was added to determine uncoupled respiration. Lastly, mitochondria complex I and III inhibitors rotenone and antimycin a were added to inhibit respiration.

Glycolytic activity was measured on a Seahorse XFe96 Analyzer (Agilent) via extracellular acidification rate (ECAR) of media, predominantly through excretion of lactate. 10 mM glucose was added to stimulate and measure glycolysis. Once reaching steady state, 1 μM oligomycin was added to inhibit ATP synthase. Lastly, 50 mM 2-Deoxyglucose (2-DG) was added to inhibit glycolysis.

### SA-β-galactosidase staining

Following 4 days of SD, myotubes were stained for SA-β-galactosidase according to the manufacturer’s instructions (Cell Signaling Technology, cat# 9860). Briefly, cells were washed 3 times with 1× PBS and fixed for 15 minutes at room temperature. After two 1× PBS washes, a β-galactosidase staining solution containing a final concentration of 1 mg/mL X-gal and a pH of 6.0 was added to the cells, the culture plate was sealed in parafilm and incubated overnight at 37°C in a dry incubator (no CO_2_). The β-galactosidase staining solution was removed and replaced with 70% glycerol. Images were taken at 10X magnification on a wide-field light microscope (Nikon Ti, Tokyo, Japan) utilizing a Nikon DS-Ri2 camera.

### BrdU proliferation assays

C2C12 myoblasts were plated at 10–20% confluence in high glucose DMEM with 10% fetal bovine serum (growth media) and allowed 24 hours to adhere to the culture dish. After 24 hours, media was changed to growth media containing 50 μM 5-bromo-2′-deoxyuridine (BrdU) (ThermoFisher, cat# B23151) and treatments. Following 6 or 68 hours of BrdU incubation, myoblasts were collected and stained with BrdU primary antibody 1:100 (Invitrogen, cat# B35130) and secondary antibody 1:500 (Invitrogen, cat# A-11001) following the manufacturer’s instruction along with 1:10,000 DAPI for 5 minutes. 7 × 7 fields were captured at 10X objective with a fully automated wide-field light microscope (Nikon Ti, Tokyo, Japan) utilizing a high-sensitivity Andor Clara CCD camera (Belfast, UK). ImageJ software was utilized to determine the total number of BrdU+ and DAPI+ cells. To determine BrdU incorporation in myotubes, cells were cultured in a 35 mm glass bottom dish (MatTek Life Sciences, cat# P35G-1.5-14-C) following the above protocol for SD. The same BrdU staining protocol was conducted but with 1:250 anti-rabbit BrdU primary antibody (Invitrogen, cat# PA5-32256) with secondary antibody (Invitrogen, cat# A21245) combined with 488 MF20 (Invitrogen, 53-6503-83). 60X objective images were captured on a wide-field light microscope (Nikon Ti, Tokyo, Japan) utilizing a high-sensitivity Andor Clara CCD camera (Belfast, UK).

### Single fiber isolation and atrophy

EDL muscle was dissected from 22-23-month-old C57Bl6 male mice and digested in a pre-warmed 2 mg/mL collagenase type I, low glucose DMEM, 1% penicillin-streptomycin solution for 90 minutes in a 37°C water bath. After digestion, EDL was transferred to a 60 mm culture plate pre-coated with 2% horse serum in warm low glucose DMEM. Single fibers were isolated by repeatedly triturating media onto the fibers. Single fibers were placed through 3 washes in 60 mm culture plates pre-coated with 2% horse serum in warm low glucose DMEM. Single fibers were finally incubated in a 37°C, 5% CO_2_ incubator in low glucose DMEM containing 20% FBS, 1% penicillin-streptomycin, and 1% chicken embryo extract. SD and MET+LEU single fibers were in media containing low glucose DMEM, 1% penicillin-streptomycin, and 1% chicken embryo extract with no FBS. Each day, fibers were imaged at 20X on an EVOS FL microscope using brightfield and transmitted settings. For each fiber, 3 images along the length of the fiber were captured and at least 5 diameter measurements were performed on each image then averaged to determine myofiber diameter.

### Human primary myoblast isolation and experimental design

Myoblasts were isolated from approximately 30–50 mg of fresh muscle tissue from a healthy 66-year-old adult male as previously described [18]. The de-identified information was part of clinical trial NCT03107884, the subjects read and signed the informed consent. The study was reviewed and approved by the University of Utah Institutional Review Board and conformed to the Declaration of Helsinki and Title 45, U.S. Code of Federal Regulations, Part 46, “Protection of Human Subjects.” Briefly, muscle tissue was minced in low glucose DMEM with 1% penicillin-streptomycin followed by 2 washes in Hank’s Balanced Salt Solution without Ca^2+^ and Mg^2+^. Tissue was then digested with collagenase II, and trypsin in 1× PBS for 30 min in a 37°C water bath. Digested muscle was then plated for 2 h in a 37°C, 5% CO_2_ incubator on a 60 mm tissue culture treated dish to adhere fibroblast and other cell populations. After 2 h, media and non-adherent cells were plated on a collagen I coated plate in low glucose DMEM with 10% FBS and 1% penicillin-streptomycin. On passage 4, myoblasts were allowed to proliferate to 95–100% confluence at which they were changed to low glucose DMEM with 2% HS, 1% penicillin-streptomycin to induce differentiation. After 8 days of differentiation, myotubes were changed to media with either 2% HS, no serum, or no serum with MET+LEU for 8 days.

### Statistical analysis

Data are reported as means ± SE with individual data points displayed. To determine differences between all treatment groups one-way ANOVA was utilized with Tukey’s post-hoc analysis. To determine differences between timepoints and treatments two-way ANOVA was utilized with Tukey’s post-hoc analysis. Unpaired sample *t*-tests were used to compare differences between two individual groups. For all statistical comparisons, significance was set at the level of *p* < 0.05. All analyses and figures were conducted with Graph Pad Prism 9 software.

## RESULTS

### Metformin + leucine combination enhanced C2C12 differentiation and inhibited myotube atrophy at low concentrations

**Figure 1:**
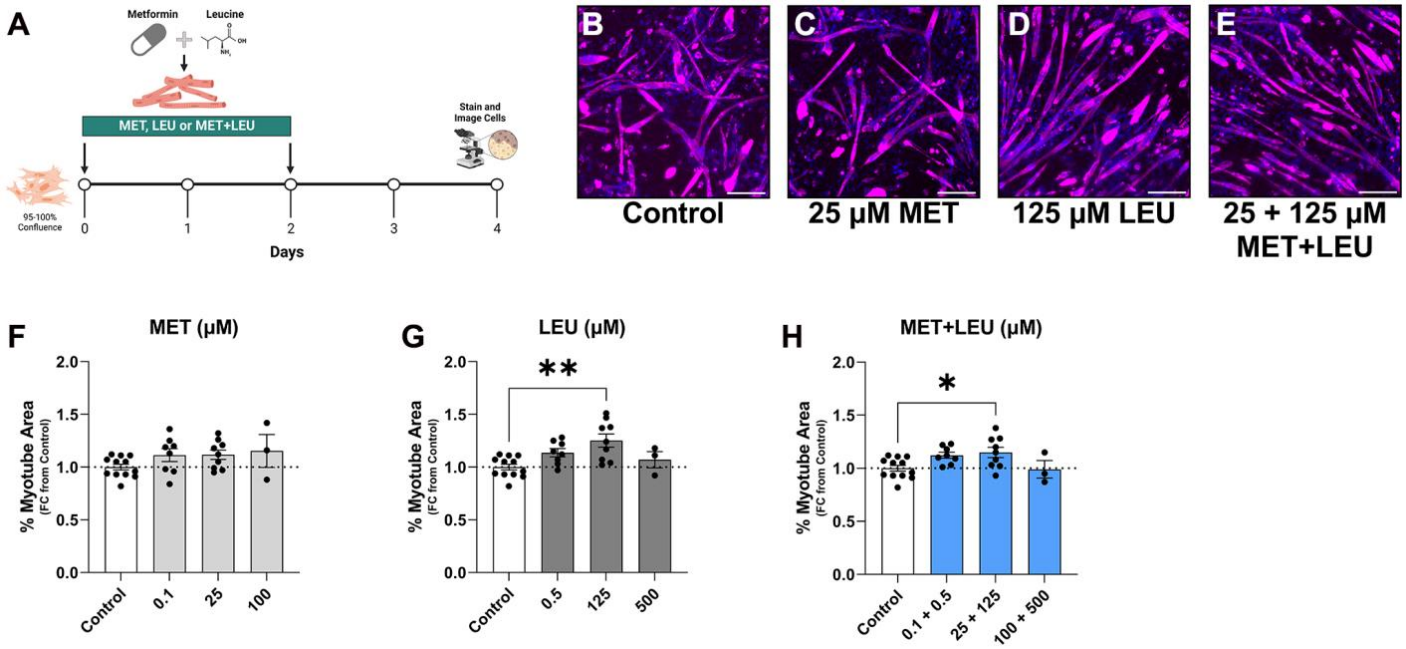
**MET+LEU increased myotube area during differentiation.** (**A**) Experimental schematic of treatments during differentiation. (**B**–**E**) Representative images of control, MET, LEU, and MET+LEU. (**F**) Myotube area after 4 days of differentiation and 0.1, 25, 100 μM MET, (**G**) 0.5, 125, and 500 μM LEU, (**H**) 0.1 + 0.5, 25 + 125, 100 + 500 μM MET+LEU. All data are represented as fold change from control. ^**^p < 0.01, ^*^p < 0.05. *N* = 3–12/group. Scale bar represents 200 μm.

**Figure 2:**
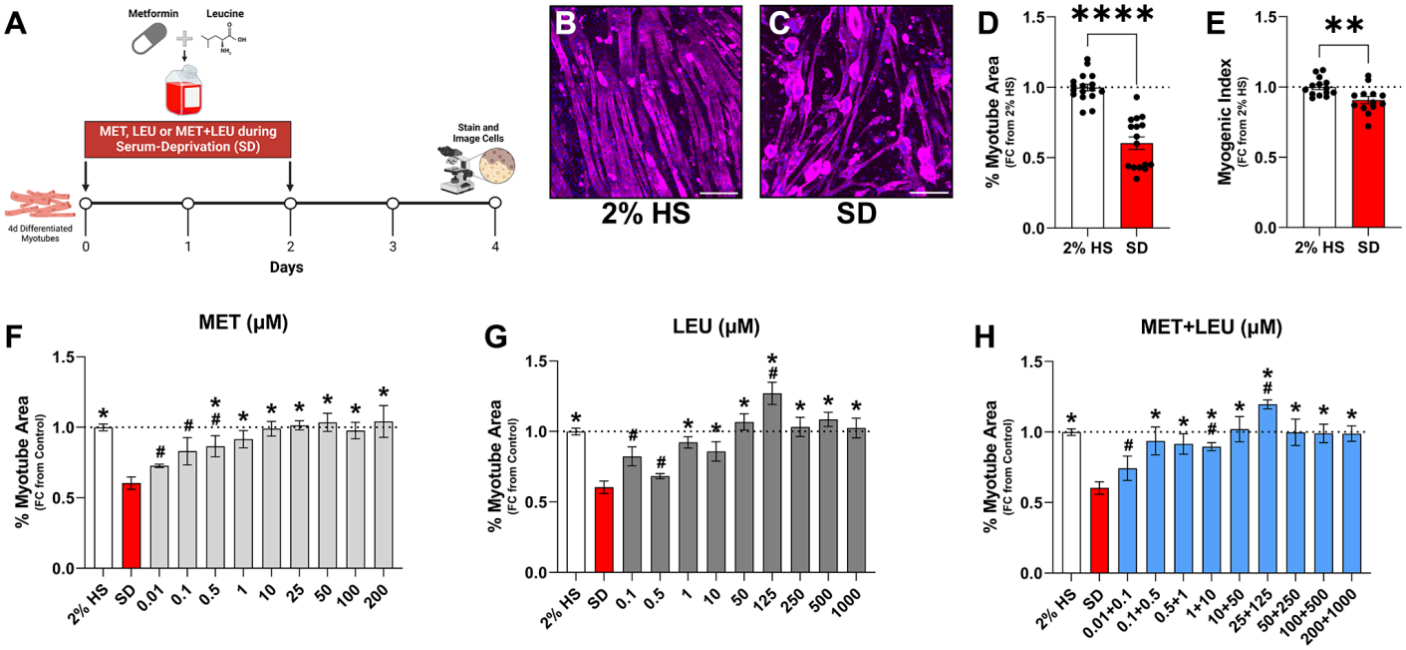
**SD caused myotube area loss that is prevented with higher concentrations of MET, LEU, and MET+LEU.** (**A**) Experimental schematic of treatments during SD. (**B**–**C**) Representative images of 2% HS and SD. (**D**) Myotube area with 2% HS or SD for 4 days. (**E**) Myogenic index (the percentage of total nuclei within myotubes) with 2% HS or SD for 4 days. Dose-response experiment with (**F**) MET, (**G**) LEU, and (**H**) MET+LEU during 4 days of SD. All data are represented as fold change from 2% HS. ^*^p < 0.05 vs. SD, ^#^*p* < 0.05 vs. 2% HS. *N* = 3–16/group. Scale bar represents 200 μm.

Following differentiation, myotube area was not altered by MET at 0.1, 25, or 100 μM (Figure 1A–1C, 1F), but was increased with 125 μM LEU (Figure 1D, 1G) and 25 + 125 μM MET+LEU (Figure 1E, 1H). To observe treatment effects during an atrophy stimulus, C2C12 myotubes were differentiated for 4 days then exposed to 4 days of SD which resulted in myotube area loss (Figure 2A–2D), and reduced myonuclear fusion (Figure 2E). Treatments prevented myotube area loss due to SD with MET concentrations of 0.5 μM to 200 μM (Figure 2F), LEU from 1 μM to 1,000 μM (Figure 2G), and MET+LEU from 0.1 + 0.5 μM to 200 + 1,000 μM (Figure 2H). 500 μM MET did not prevent myotube atrophy and beyond this concentration was cytotoxic. Contrarily, concentrations of up to 30 mM LEU were able to prevent myotube area loss due to SD with no cytotoxicity (data not shown). 10, 25, 50, and 100 μM MET increased myonuclei fusion vs. SD (Supplementary Figure 1A), where LEU and MET+LEU did not (Supplementary Figure 1B, 1C). At a low concentration (0.1 + 0.5 μM), MET+LEU was able to prevent myotube area loss where individual MET or LEU treatments did not, indicative of the potential synergy of MET and LEU at low concentrations (Figure 2F–2H).

### Low concentration metformin + leucine uniquely altered myotube area and transcriptional profiles during serum-deprivation

**Figure 3:**
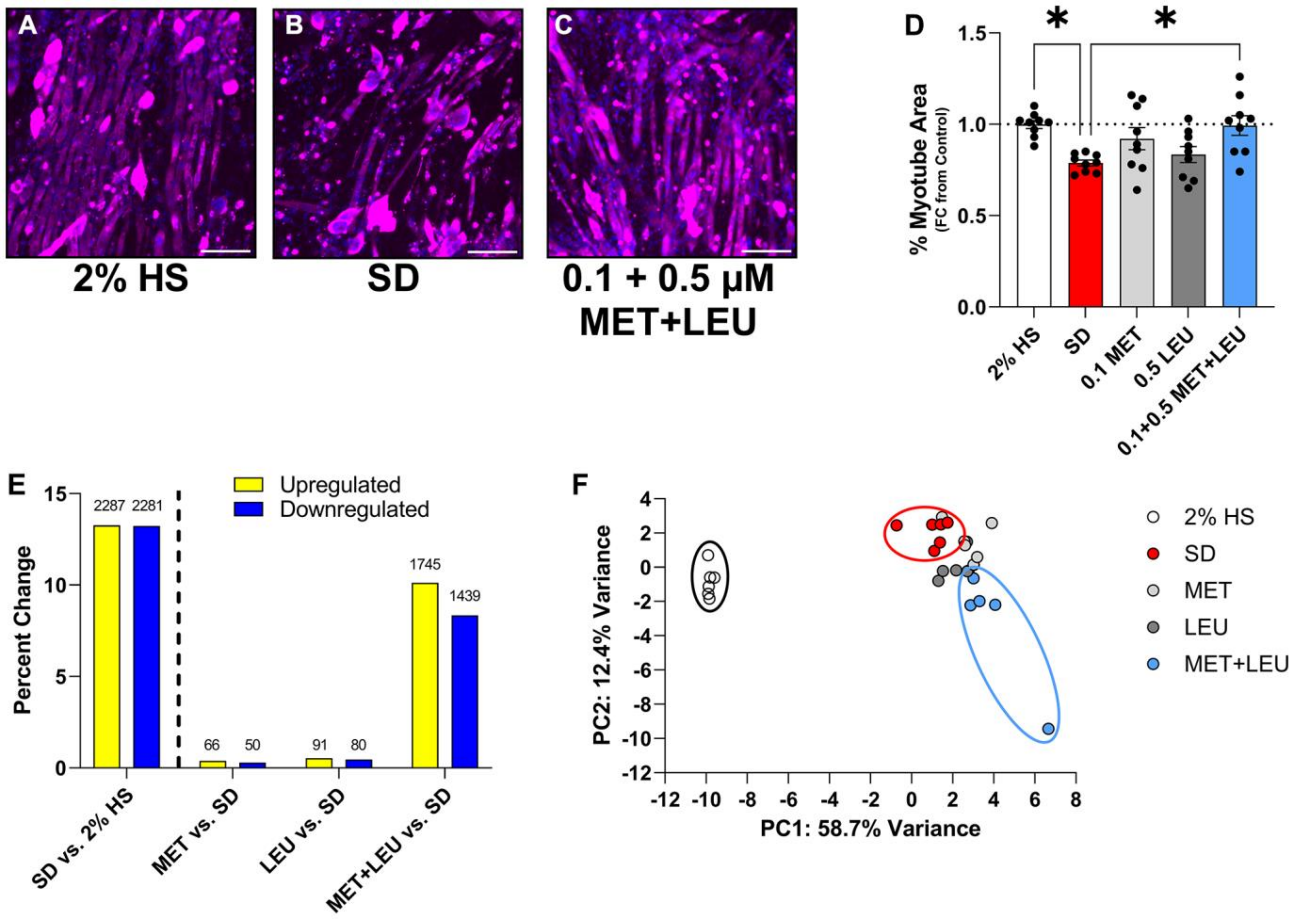
**Low concentration MET+LEU uniquely prevents myotube atrophy and alters transcriptional profile during SD.** (**A**–**C**) Representative images of 2% HS, SD, and 0.1 + 0.5 μM MET+LEU. (**D**) Myotube area observing the effects of low concentration MET, LEU, and MET+LEU after 4 days of SD as a fold change from 2% HS. (**E**) The percent change in upregulated and downregulated transcripts in SD vs. 2% HS, MET vs. SD, LEU vs. SD, and MET+LEU vs. SD with the number of transcripts changed above each bar. (**F**) Principle component analysis based on the top 500 transcripts changed with 2% HS, SD, and low concentration MET, LEU, and MET+LEU. ^*^p < 0.05. *N* = 9/group for (**A**–**D**), *N* = 6/group for (**E** and **F**). Scale bar represents 200 μm.

When comparing 0.1 μM MET, 0.5 μM LEU, and 0.1 + 0.5 μM MET+LEU, only MET+LEU prevented myotube area loss during SD (Figure 3A–3D). Transcriptional profiling revealed that SD caused upregulation of 2,287 (~13%) and downregulation of 2,281 (~13%) transcripts vs. 2% HS (Figure 3E). Compared to SD, MET and LEU altered <1% of transcripts where MET+LEU caused upregulation of 1,745 (~10%) and downregulation of 1,439 (~8%) transcripts (Figure 3E). Based on the top 1000 differentially expressed genes, PCA plot displayed MET+LEU treated myotubes clustered separately from SD (Figure 3F). These data support that SD caused transcriptional changes compared to serum-containing controls (2% HS) and that at low concentrations are uniquely altered by the MET+LEU combination.

We also tested the combination of metformin, leucine, and sildenafil (MET+LEU+SILD) since the triple combination has shown to further synergize metformin and leucine effects in hepatocytes and macrophages [20]. However, during SD, sildenafil alone (Supplementary Figure 2A) or in combination with MET+LEU (Supplementary Figure 2B) did not affect myotube area loss. It is possible that due to sildenafil action via phosphodiesterase type 5 (PDE5) inhibition leading to increased cyclic GMP [21], and thus vasodilation, that triple combination treatment would have more robust effects *in vivo* through vascular endothelial cell function. Therefore, we moved forward to examine the unique mechanisms of MET+LEU. To expand the generalizability of MET+LEU, we tested the cocktail on other atrophy-inducing stimuli. Beyond serum deprivation, MET+LEU was able to prevent inflammatory, TNF-α-induced atrophy (Supplementary Figure 3A) and partially reduced fatty acid, palmitate-induced atrophy (Supplementary Figure 3B).

### Proteostasis is maintained with metformin + leucine during serum-deprivation independent of AMPKα and mTORC1 signaling

**Figure 4:**
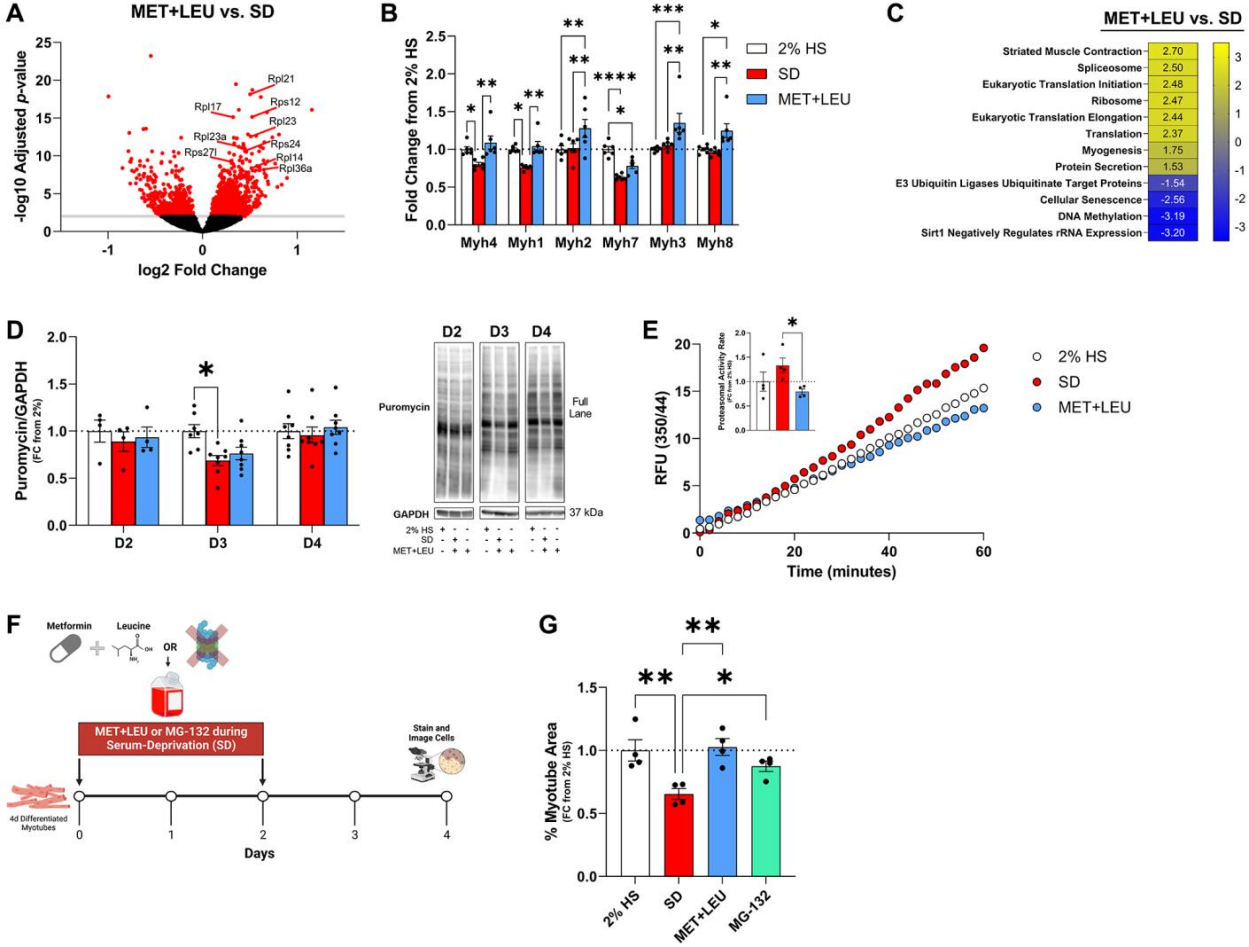
**MET+LEU maintained proteostasis during SD.** (**A**) Volcano plot of changed transcripts with MET+LEU vs. SD with a significance cut of -log10Adjusted *p*-value of 2, highlighting ribosomal subunit genes. (**B**) Myosin heavy chain genes from RNA-sequencing in 2% HS, SD, and MET+LEU. (**C**) Pathways altered with MET+LEU vs. SD related to proteostasis, numbers are representative of normalized enrichment scores (NES). (**D**) Puromycin incorporation after 2-days (D2), 3-days (D3), and 4-days (D4) of SD and representative western blot. (**E**) Proteasome activity assay results represented as relative fluorescent units, (E inset) calculated proteasomal activity rate. (**F**) Experimental schematic of 16.8 μM MG-132, or 0.1 + 0.5 μM MET+LEU treatment with SD. (**G**) Myotube area during 4 days SD, MET+LEU, or MG-132. ^****^p < 0.0001, ^**^p < 0.01, ^*^p < 0.05. *N* = 6/group for (**A**–**D**), *N* = 4/group for (**E** and **F**).

In MET+LEU vs. SD, the most differentially expressed transcripts included ribosomal biogenesis and ribosomal subunit genes (Figure 4A). Additionally, SD reduced myosin heavy chain transcripts vs. 2% HS that were alleviated with MET+LEU or increased beyond 2% HS (Figure 4B). In MET+LEU vs. SD, we noted important pathways related to translation, muscle function/structure, and myogenesis were upregulated while protein breakdown pathways were downregulated (Figure 4C). Following 3-days of SD, protein synthesis was reduced in SD vs. 2% HS (Figure 4D), which did not occur with MET+LEU treatment. Following 4 days of SD, proteasome activity is reduced in MET+LEU vs. SD (Figure 4E). To observe the contribution of proteasome activity on myotube atrophy during SD, we gave increasing doses of the proteasome inhibitor, MG-132 (Supplementary Figure 4). Proteasome activity inhibition resulted in prevention of myotube area loss compared to SD (Figure 4F, 4G) and comparable to MET+LEU. While AMPKα and mTORC1 signaling are perturbed by SD, there were no differences with MET+LEU vs. SD on AMPKα acute (15 to 480 min; Supplementary Figure 5A–5D) or chronic (2 to 4 days; Supplementary Figure 5E–5H) signaling or mTORC1 acute (Supplementary Figure 5I–5L) or chronic (Supplementary Figure 5M–5P) signaling.

### Autophagy, apoptosis, endoplasmic reticulum stress, and mitochondria dysfunction are unaltered with metformin + leucine compared to serum-deprivation

We observed additional pathways related to proteostasis to determine the mechanistic contributions of MET+LEU in myotube atrophy prevention. Markers of autophagy and apoptosis were not acutely or chronically altered with MET+LEU vs. SD (Supplementary Figure 6A–6R). Similarly, markers of ER stress were not acutely or chronically changed with MET+LEU vs. SD (Supplementary Figure 7A–7M).

Following 4 days of SD, transcriptional profiling supported that pathways related to mitochondrial biogenesis and function were upregulated in MET+LEU vs. SD, where glycolytic-related pathways were reduced (Supplementary Figure 8A). Therefore, we performed extensive phenotyping on whole cell and isolated mitochondrial content and function. Mitochondria complex content (Supplementary Figure 8B, 8C), isolated mitochondria maximal respiration (Supplementary Figure 8D), isolated mitochondria efficiency (Supplementary Figure 8E), whole cell oxygen consumption (Supplementary Figure 8F), whole cell glycolytic activity (Supplementary Figure 8G), and lactate production (Supplementary Figure 8H) were unchanged in MET+LEU vs. SD. Additionally, the ROS markers in isolated mitochondria of H_2_O_2_ production and emission (Supplementary Figure 8I), H_2_O_2_ leak percentage (Supplementary Figure 8J), in whole muscle cell superoxide dismutase 2 (SOD2; Supplementary Figure 8K), and lipid peroxidation (4-HNE; Supplementary Figure 8L) were unaffected with MET+LEU vs. SD.

### Metformin + leucine treatment reduced inflammatory and cellular senescence markers

**Figure 5:**
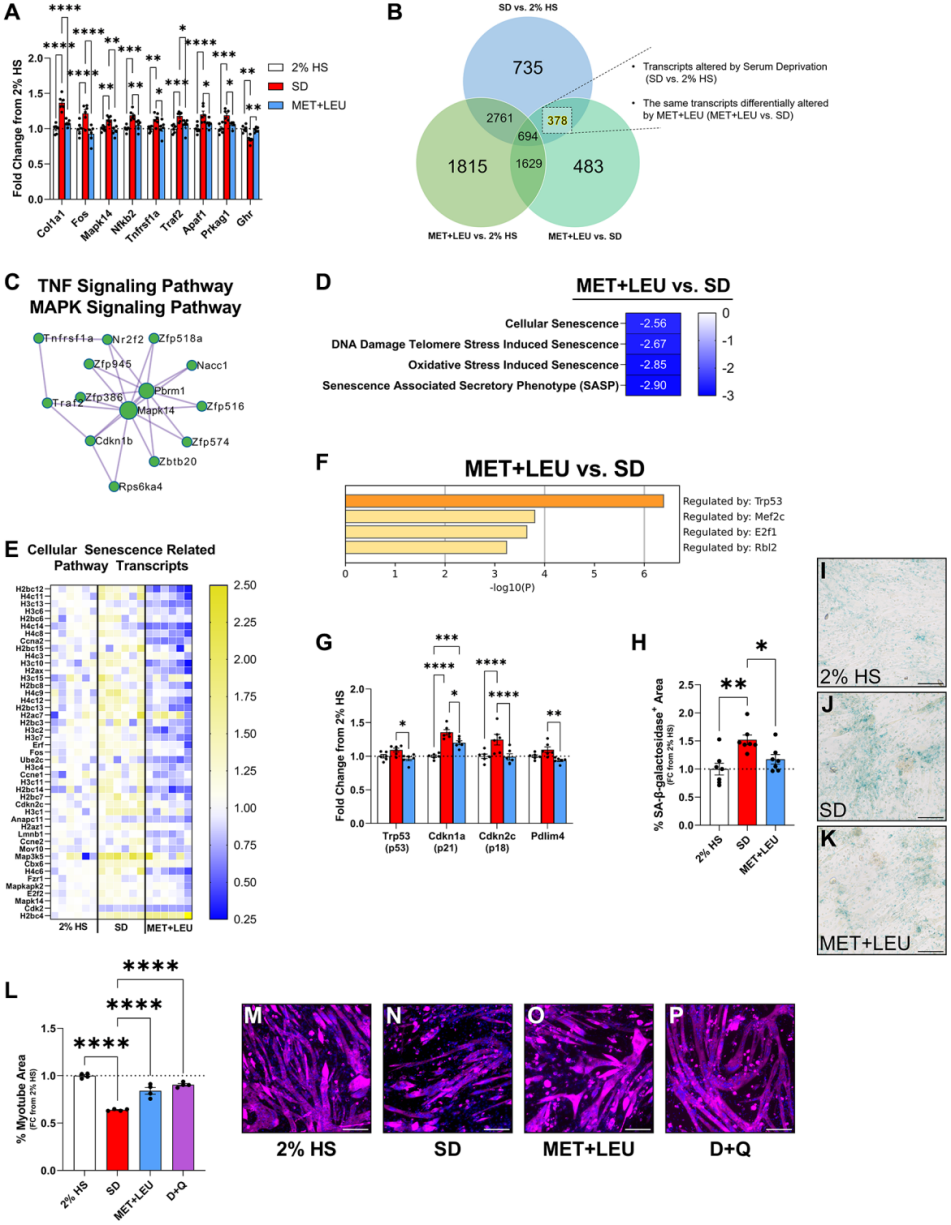
**MET+LEU reduced inflammatory transcriptional profile and markers of cellular senescence.** (**A**) Inflammation-related genes from RNA-sequencing in 2% HS, SD, and MET+LEU as fold change from 2% HS. (**B**) Venn diagram of how 378 genes altered with SD and reversed with MET+LEU was determined. (**C**) Protein-protein interaction map displaying transcripts reversed with MET+LEU was in the interconnected signaling networks of TNF and MAPK pathways. (**D**) Pathways altered with MET+LEU vs. SD related to cellular senescence, numbers are representative of NES. (**E**) Heatmap of gene expression represented as a fold change from 2% HS of genes within the cellular senescence-related pathways. (**F**) TRRUST enrichment analysis of predicted regulators of transcriptional changes with MET+LEU vs. SD in reversed genes. (**G**) Cellular senescent-related genes from RNA-sequencing in 2% HS, SD, and MET+LEU as fold change from 2% HS. (**H**) SA-β-galactosidase area analysis in 2% HS, SD, and MET+LEU. (**I**–**K**) Representative images of SA-β-galactosidase staining. (**L**) Myotube area during 4 days SD, MET+LEU or 62.5 nM dasatinib + 12.5 μM quercetin (D+Q). (**M**–**P**) Representative images for analyzing myotube area. All data are after 4 days of SD. ^****^p < 0.0001, ^***^p < 0.001, ^**^p < 0.01, ^*^p < 0.05. *N* = 6/group for (**A**–**G**), *N* = 7/group for (**H**–**K**). *N* = 4/group for (**L**–**P**). Scale bar represents 200 μm.

Following 4 days of SD, a notable observation was that transcripts related to inflammation in MET+LEU vs. SD were markedly decreased (Figure 5A). After determining the differentially regulated genes that were altered with SD vs. 2% HS and reversed with MET+LEU (Figure 5B), we discovered there were 378 genes reversed to 2% HS levels with MET+LEU during SD, and we performed a separate analysis on these transcripts. Within the reversed genes, protein-protein interaction enrichment analysis identified TNF signaling (Log10(P) = −6.1) and MAPK signaling pathways (Log10(P) = −4.4) as altered networks (Figure 5C). Unbiased pathway analysis displayed decreased cellular senescence pathways (Figure 5D) and transcripts related to cellular senescence when comparing MET+LEU vs. SD (Figure 5E). Interestingly, senescence pathways were reduced with LEU vs. SD, and with MET+LEU vs. LEU suggesting LEU has anti-senescence properties that are synergized with MET (Supplementary Figure 9A). In the 378 reversed genes, enrichment analysis defined p53 as a predicted dominant regulator of gene expression (Figure 5F) in MET+LEU vs. SD. The cellular senescence-related transcripts Trp53 (p53), Cdkn1a (p21), Cdkn2c (p18), and pdlim4 were all reduced with MET+LEU vs. SD (Figure 5G). We followed up with SA-β-Galactosidase staining and noted an increase in SA-β-Galactosidase in SD vs. 2% HS but decreased to 2% HS levels by MET+LEU (Figure 5H–5K). Senolytic treatment with dasatinib + quercetin (D+Q) during SD prevented myotube atrophy similar to MET+LEU (Figure 5L–5P), at varying doses (Supplementary Figure 9B).

### Cell cycle transcriptional pathways increased by SD were reversed with metformin + leucine but MET+LEU did not alter cellular proliferation

Enrichment plots for GSEA Hallmark pathways in the 378 reversed genes discovered only two significantly influenced pathways: G2M Checkpoint and E2F Targets. Both G2M Checkpoint and E2F Target pathways are reduced with MET+LEU vs. SD and increased in SD vs. 2% HS (Supplementary Figure 10A). Additionally, to G2-M Checkpoint and E2F Target transcripts (Supplementary Figure 10B), genes important in cell cycle function such as cyclins and cyclin-dependent kinases were reduced with MET+LEU vs. SD (Supplementary Figure 10C). Thus, we conducted BrdU labeling proliferation experiments in C2C12 myoblasts. We explored myoblast proliferation with acute (6-hour, Supplementary Figure 10D) and chronic (68-hour, Supplementary Figure 10E) MET+LEU treatment. The percentage of proliferating cells was not different in either condition with MET, LEU, or MET+LEU treatment (Supplementary Figure 10F). Our transcriptional data thus eluded that the terminally differentiated myotubes may have attempted to activate cell cycle programs, and therefore we examined if these myotubes may be expressing BrdU, confirming an attempt of myonuclei to proliferate within the myotubes (Supplementary Figure 10G). Indeed, some myonuclei within myotubes demonstrated BrdU incorporation (Supplementary Figure 10H), however, this was observed similarly across groups elucidating that it was not a phenomenon driven by SD. These data could be due to late myoblast fusion events but may also agree with recent work by Borowik et al. supporting that myonuclei are indeed not post-mitotic [22].

### Metformin + leucine prevented atrophy in isolated aged mouse myofibers and improved myoblast fusion in primary human muscle cells from an aged donor

**Figure 6:**
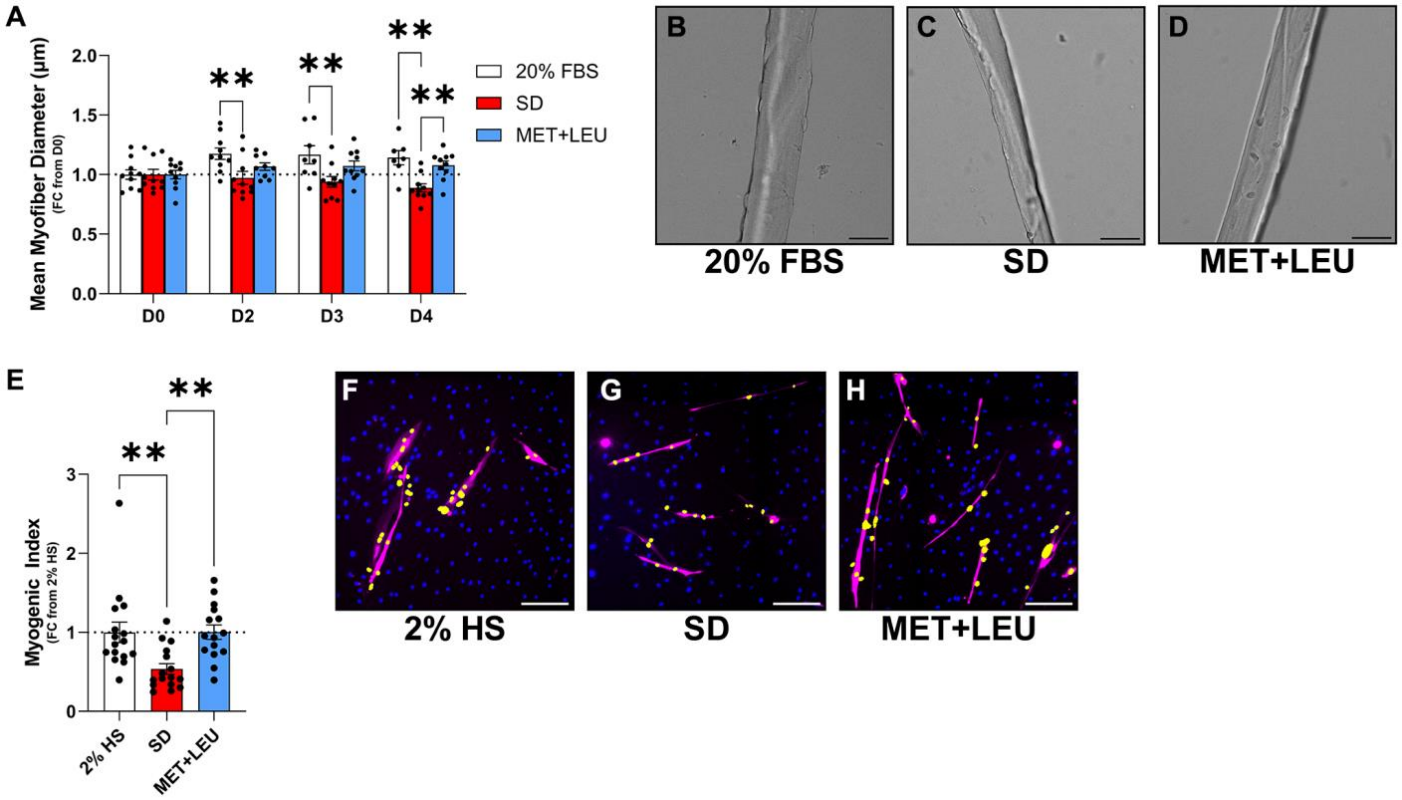
**MET+LEU prevented aged myofiber atrophy and improved myonuclei fusion in primary human myotubes from an aged donor.** (**A**) Mean myofiber diameter in single isolated myofibers from aged mice with 0, 2, 3, and 4 days of either 2% HS, SD, or MET+LEU during SD. (**B**–**D**) Representative images of isolated myofibers with different treatments. (**E**) Myogenic index in primary myotubes from an aged donor following 8-days differentiation then 8-days of 2% HS, SD, or MET+LEU during SD represented as fold change from 2% HS. (**F**–**H**) Representative images of primary myotubes, yellow label indicates nuclei fused into myotubes. ^**^p < 0.01, *N* = 7–10/group for (**A**–**D**) and *N* = 15–16/group for (**E**–**H**). Scale bar represents 200 μm.

To support the muscle cell-specific translational relevance of MET+LEU, we isolated single myofibers from EDL muscle of aged mice (22–23 months) and cultured them in 20% FBS, SD, or MET+LEU during SD for 4 days. Two, 3, and 4 days of culture in SD media caused a decrease in myofiber diameter compared to fibers cultured in 20% FBS media (Figure 6A–6C). After 4 days in SD, MET+LEU prevented myofiber atrophy vs. SD alone (Figure 6A, 6D). In primary human myoblasts, MET+LEU prevented a decrease in myogenic index caused by SD (Figure 6E–6H) but did not prevent the decrease in myotube area (Fold change from control- SD: 0.36 ± 0.02, MET+LEU: 0.45 ± 0.03 mean ± SEM).

## DISCUSSION

**Figure 7:**
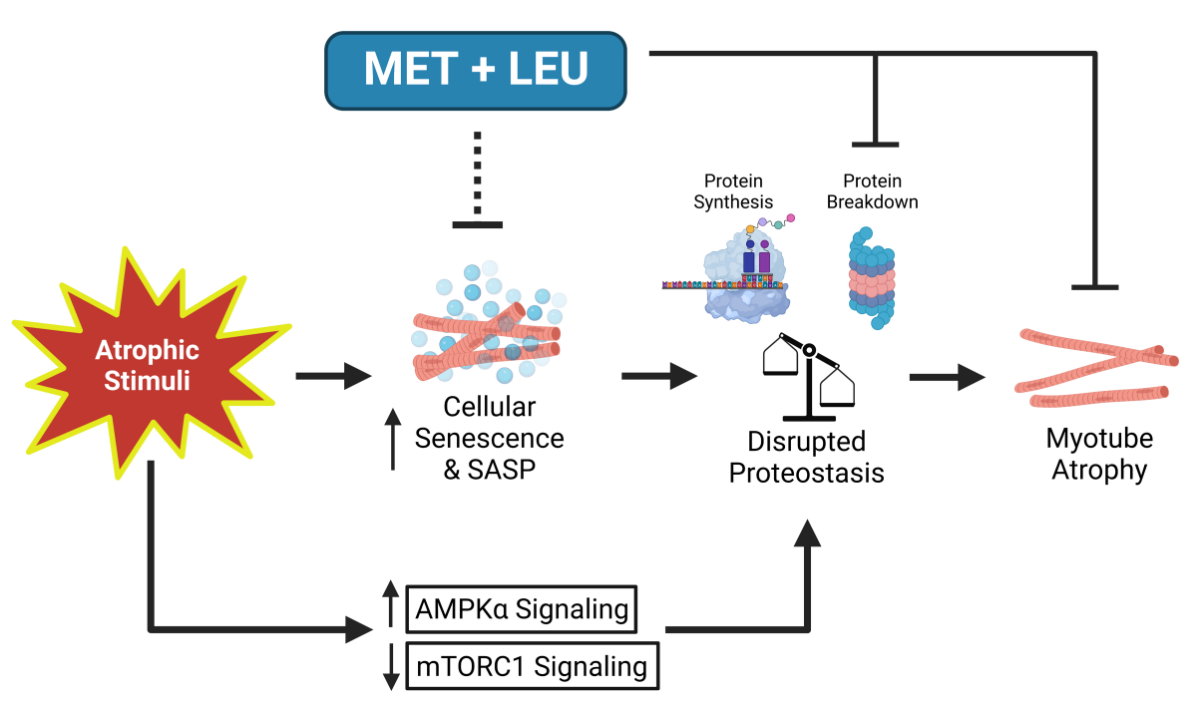
**Summary schematic of MET+LEU effects during SD in muscle cells.** Following atrophic stimuli (serum deprivation (SD)), acutely (within 4 hours) AMPKα signaling increased while mTORC1 signaling decreased. With chronic (4 days) serum deprivation, cellular senescence and inflammation related to the senescence-associated secretory phenotype (SASP) are increased. These contribute to disrupted proteostasis (decreased protein synthesis and increased protein breakdown) that eventually caused myotube atrophy. MET+LEU appears to inhibit SD-induced cellular senescence and inflammatory-related SASP which may have caused the prevention in proteasome activity observed with MET+LEU, independent of AMPKα and mTORC1 signaling. MET+LEU was also effective in preventing myotube atrophy following other modes of atrophic stimuli (TNF-α- and lipid-induced) while also reducing atrophy *ex vivo* in aged myofibers and preventing a decline in aged human primary myotube myonuclei fusion.

The major findings from this study were that a low-dose combination of metformin and leucine (MET+LEU) prevented serum deprivation-mediated skeletal muscle cellular senescence and inflammatory pathways, restored proteostasis, and prevented myotube atrophy. We also observed that MET+LEU was effective to reduce atrophy across other cell culture models of muscle atrophy. Finally, MET+LEU inhibited single myofiber atrophy from aged mice and increased myonuclei fusion in primary muscle cells from an aged donor supporting the translational and aging relevance of the muscle-specific role of MET+LEU. Together, these data suggest that in skeletal muscle cells, low-dose MET+LEU was able to prevent myotube atrophy and this may be by reducing cellular senescence thereby reversing heightened inflammatory pathways and maintaining proteostasis (Figure 7).

Senescent cells accumulate with advanced age and become problematic as their secretory profile promotes inflammation and tissue dysfunction [23]. While senescence is defined by cellular growth arrest, a senescence-like phenotype has also been observed in post-mitotic cells [24–26], contributing to SASP-driven tissue dysfunction. In skeletal muscle tissue, senescent cells are detected in mononuclear populations (e.g., fibroadipogenic progenitors, macrophages) and recently amongst myonuclei from isolated myofibers in aged mice [10]. Interestingly, in myofibers from aged mice the common senescent marker, p16, is undetectable, but populations of high p21-expressing nuclei are evidently expressed [10]. Similarly, in skeletal muscle from older compared to younger individuals, p16 was lowly expressed yet p21 was markedly elevated [27]. Intriguingly, global p21 overexpression was capable to impair muscle function, promote muscle inflammation and fibrosis, and reduce myofiber size [8] suggesting that p21 may be a reliable marker to denote cellular senescence in mature myofibers undergoing atrophy. In our experiments following 4 days of an atrophy stimulus, myotubes had increased transcriptional levels of p21 and were similarly absent of changes in p16 expression. Importantly, we found that MET+LEU impeded the increase in p21 transcription, decreased inflammatory pathways, and reduced the senescent marker SA-β-galactosidase during muscle atrophy. Future studies examining the role of MET+LEU to prevent senescence independent of atrophy, such as with chemical inducers of senescence (H_2_O_2_ or Palbociclib) may be needed to determine a direct role of MET+LEU alleviating senescence. However, our results suggest that MET+LEU partly operates through cellular senescent pathways *in vitro* and is linked to myofiber atrophy *ex vivo* in mature fibers.

Metformin has been reported to prevent cellular senescence in many types of cells such as fibroblasts [11, 13, 28], myoblasts [13], and vascular smooth muscle cells [17]. Mechanistically, the effect of metformin on senescence has been described to be at least through AMPKα [16, 28], though other pathways such as autophagy [17], ROS [14], inflammation [11], or ER stress [13] have been involved. The role of leucine to target senescent cells is less understood [29]. We suggest that leucine may have a supportive role in enhancing the protective effects of metformin on senescent cells since LEU alone reduced cellular senescence pathways and MET+LEU combination further reduced senescence and inflammatory pathways than either alone. Surprisingly, MET+LEU influence on senescence and inflammatory pathways was independent of AMPKα. In agreement with our prior work, MET+LEU treatment in aged mice during hindlimb unloading similarly rescued inflammatory-related transcriptional pathways independent of AMPKα [4]. Likewise, in another study, metformin effects on senescence were shown to be through NF-kappaB activation independent of AMPKα signaling [11] and we too noted increased transcripts related to NFKB signaling with the MET+LEU combination. Therefore, metformin synergized by leucine regulates cellular senescence in myofibers possibly through an NFKB mechanism, and is independent of AMPKα signaling. Interestingly, we found that, similar to MET+LEU, the senolytic cocktail, dasatinib+quercetin, reduced myotube atrophy, suggesting that the mature myofiber size may be partly regulated by cellular senescence.

Cellular senescence occurs during aging and in parallel with muscle atrophy. However, the contribution of cellular senescence to muscle atrophy is only beginning to be examined [8, 9]. Whole body p21 overexpression resulted in increased cellular senescence and muscle atrophy [8], and aged humans have an increased percentage of gamma H2A histone family member X (γH2AX) positive satellite cells [30]. Further, depletion of SA-β-galactosidase-positive macrophages in aged mice improved muscle regeneration [31], and introducing senescent cells to young mice during muscle recovery following chemical injury creates an aged-like microenvironment and impairs regeneration [32, 33]. Together, these data indicate that interstitial cells and the cellular environment that are important in muscle remodeling are influenced by senescence. The effect of MET+LEU to avert myotube atrophy may operate through senescent cell removal, leading to reduced inflammatory signaling and thus balanced proteostasis. Proteostasis and cellular senescence are likely closely integrated. For example, proteostasis is dysregulated in senescent human primary fibroblasts [34], and SASP factors have roles in modulating proteostasis. While not fully explored in this study, our data infers a possible relationship between disrupted proteostasis in muscle atrophy and cellular senescence.

Lastly, we explored candidate atrophy mechanisms related to, oxidative phosphorylation, AMPKα and mTORC1 signaling, autophagy, apoptosis, ER stress, and cell cycling, due to positive hits in our transcriptional data sets with MET+LEU. But when examined functionally or at the protein level in muscle cells they were not consistent with the transcriptomic data and therefore did not explain atrophy prevention caused by the MET+LEU combination. However, we cannot completely rule out these mechanisms for explaining the pleiotropic effects of MET+LEU. For example, it is possible MET+LEU altered oxidative phosphorylation and/or ROS emission earlier in our experimental timeline during serum deprivation thus we might have missed the window of rescue. This could be the case since impaired mitochondrial function and ROS are known mediators of muscle atrophy [35], low-concentration metformin can improve mitochondrial function [36], and that oxidative stress primes aged satellite cells to become senescence [32].

In summary, MET+LEU treatment prevented myofiber and myotube area loss, maintained proteostasis, and decreased markers of cellular senescence. MET+LEU also increased myonuclei fusion in primary human myotubes from an aged donor. The effects of MET+LEU in muscle cells coincided with an improved inflammatory transcriptional profile. In conclusion, this study provides evidence of a possible link between cellular senescence and disrupted proteostasis that is targeted by MET+LEU in muscle cells to reverse the muscle atrophy phenotype.

## Supplementary Materials

Supplementary Figures
