# Supplementary material for: Cellular senescence and disrupted proteostasis induced by myotube atrophy are prevented with low-dose metformin and leucine cocktail
Source: Aging (Albany NY). 2023 Mar 20;15(6):1808–32. doi: 10.18632/aging.204600 (PMC10085594; doi:10.18632/aging.204600)
Supplement: Supplementary Figures [file aging-15-204600-s001.pdf]

SUPPLEMENTARY FIGURES

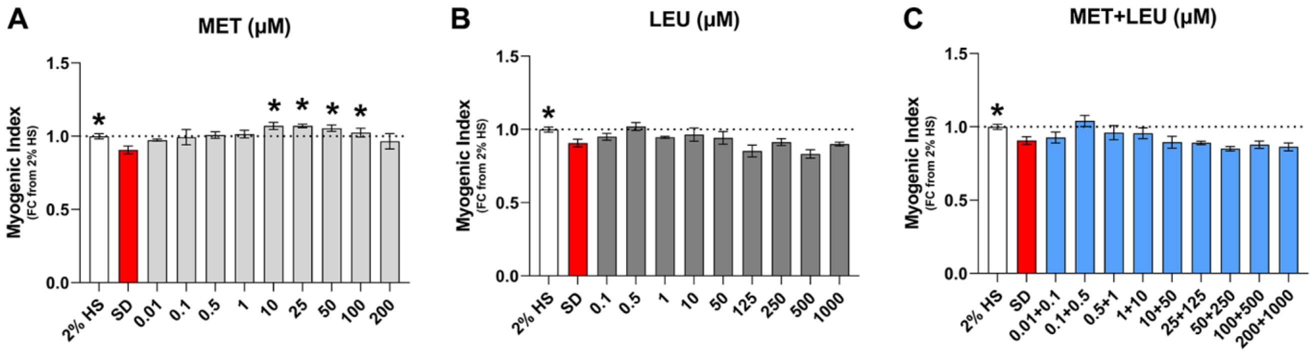

Supplementary Figure 1. MET, but not LEU or MET+LEU alter myonuclei fusion during SD. After 4-days of SD, myogenic index of differing doses of (**A**) MET, (**B**) LEU, and (**C**) MET+LEU. $^{*}p < 0.05$ vs. SD, N = 3–16/group.

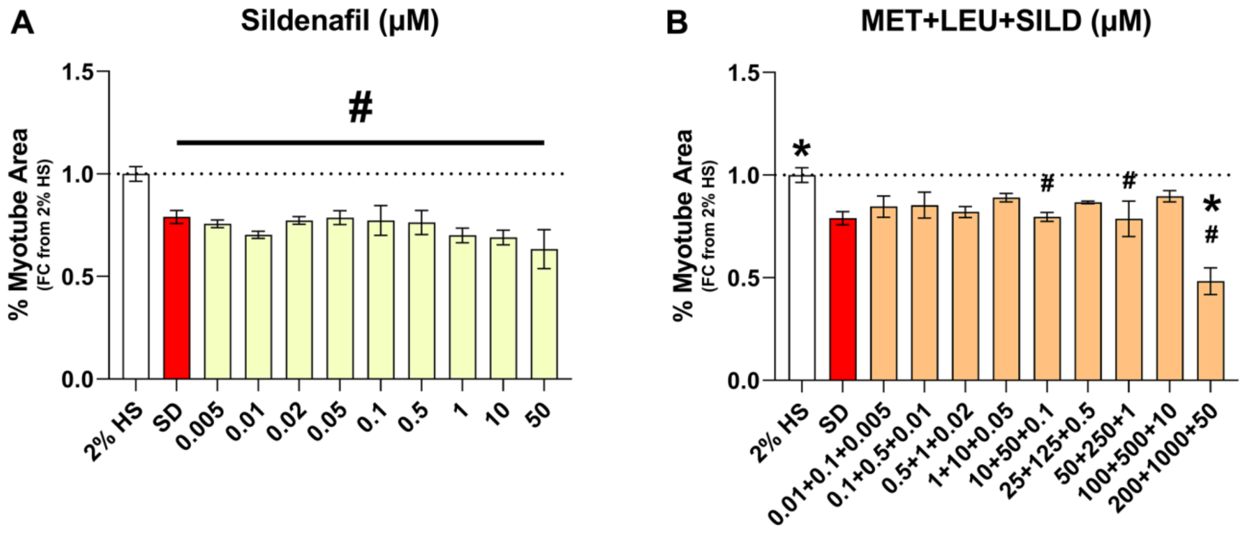

Supplementary Figure 2. Sildenafil or MET+LEU+SILD combination does not prevent myotube atrophy during SD. After 4-days of SD, myotube area with differing doses of (**A**) sildenafil alone or (**B**) metformin + leucine + sildenafil (MET+LEU+SILD) triple combination. $^{*}p < 0.05$ vs. SD, $^{\#}p < 0.05$ vs. 2% HS. N = 3/group.

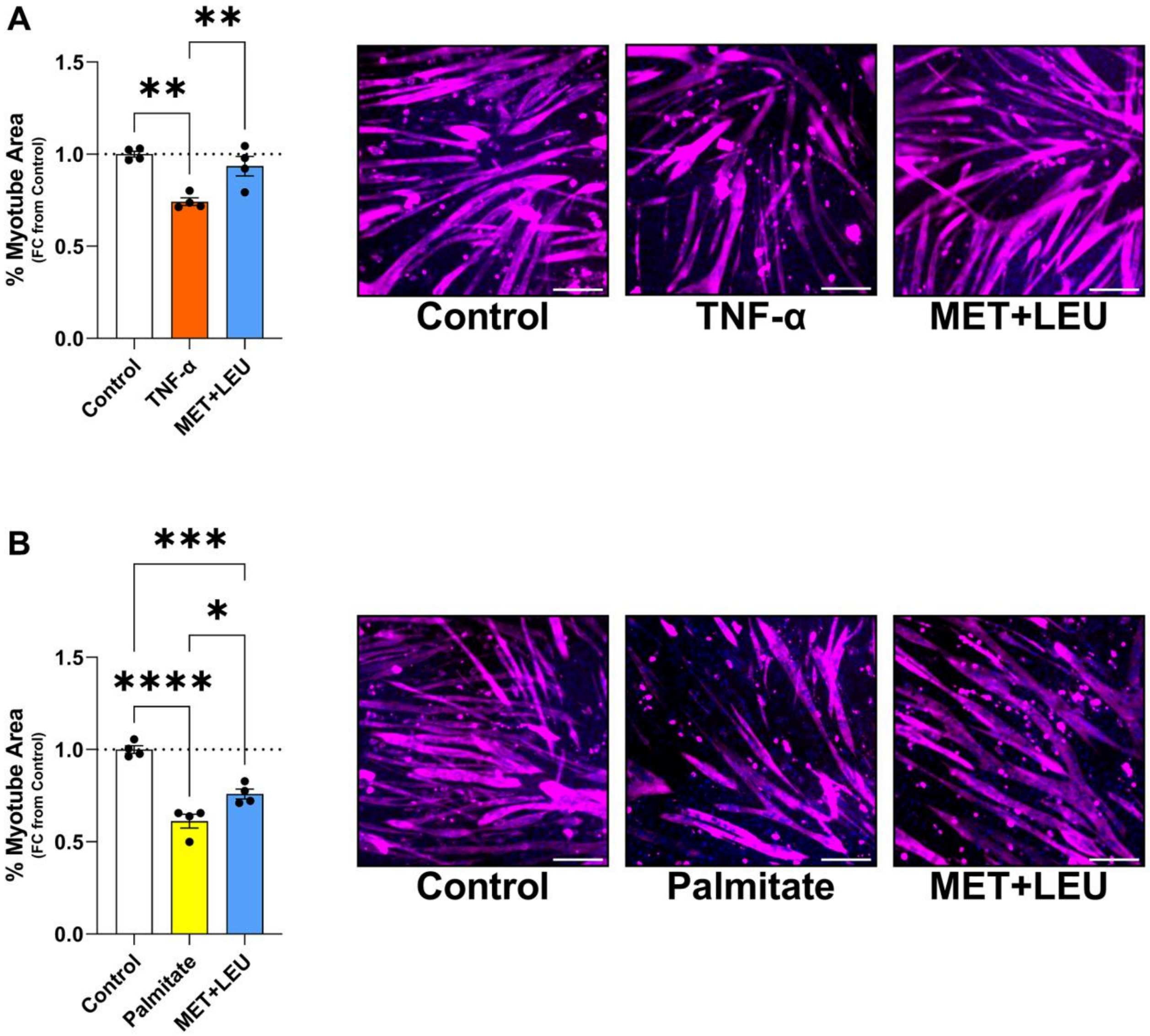

Supplementary Figure 3. MET+LEU during inflammatory- and fatty acid-induced atrophy. After 3 days of 100 ng/mL TNF-α with or without MET+LEU (**A**). After 1 day of 0.75 mM palmitate with or without MET+LEU (**B**). *N* = 4/group.

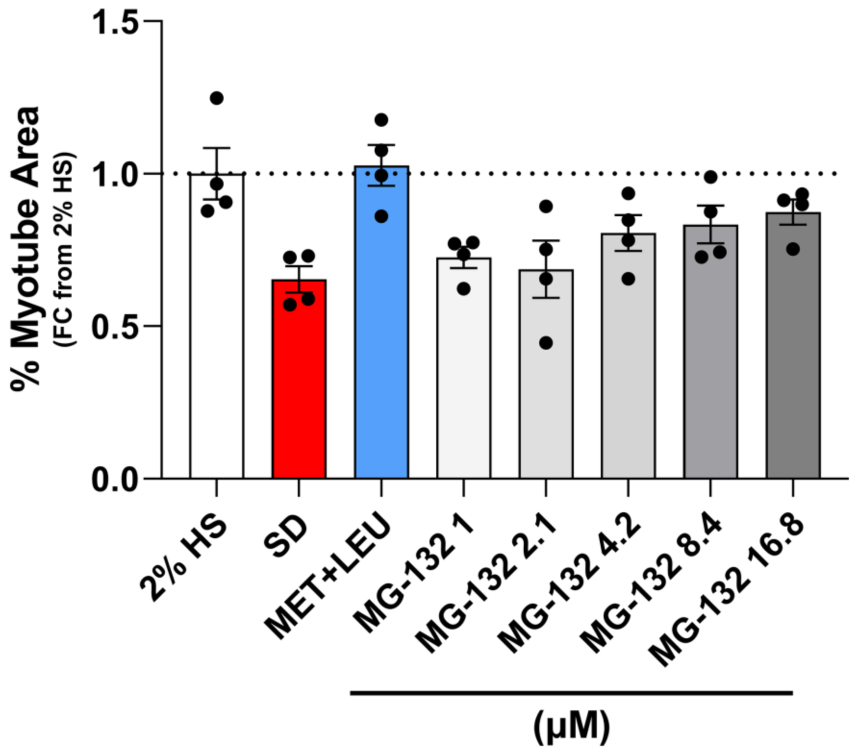

Supplementary Figure 4. Proteasome inhibition prevents myotube atrophy during SD in a dose dependent manner. After 4-days of SD, myotube area with 0.1 + 0.5 µM MET+LEU, or doses of 1, 2.1, 4.2, 8.4, and 16.8 µM MG-132 (proteasome inhibitor). *N* = 4/group.

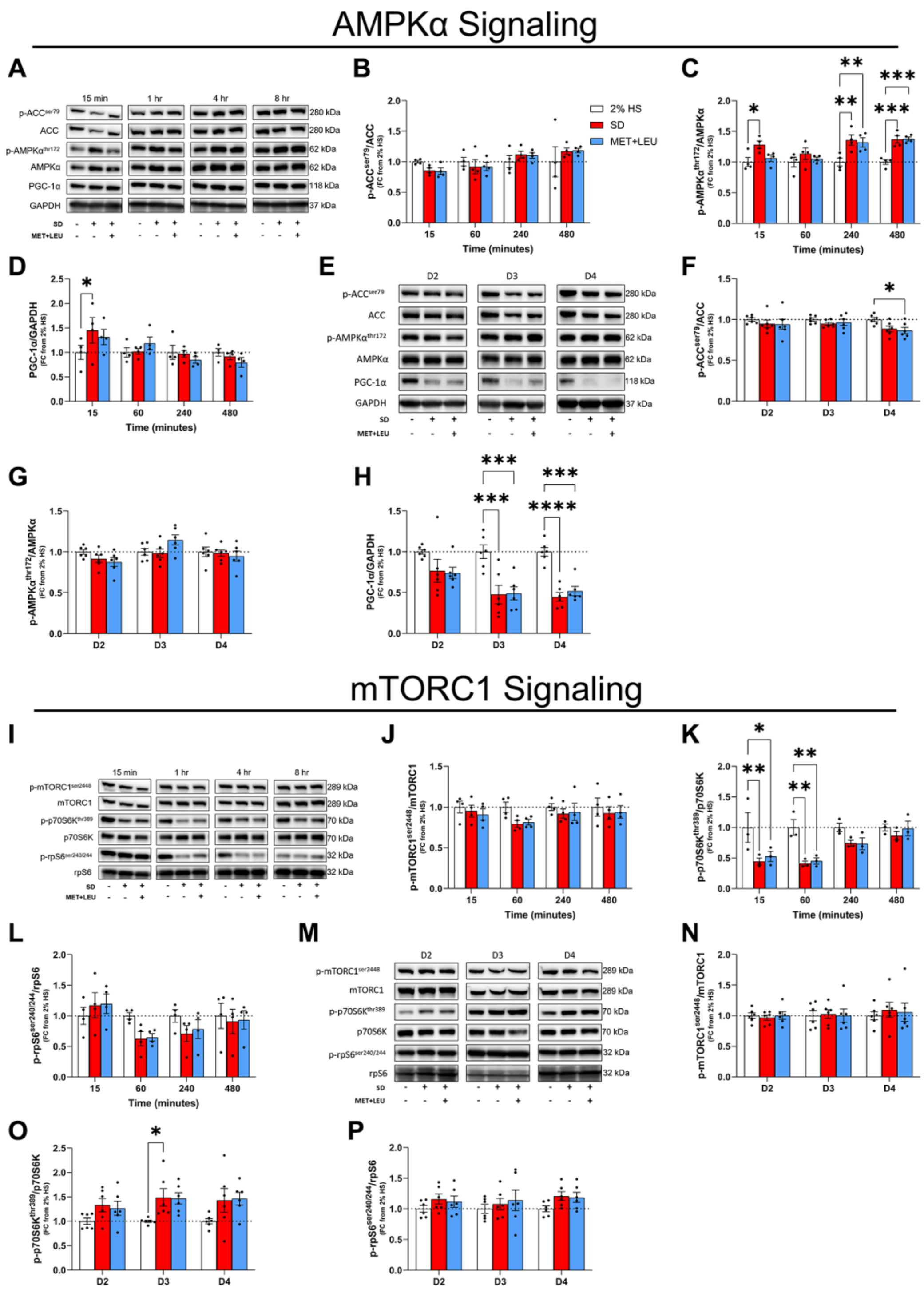

Supplementary Figure 5. Acute and chronic AMPKα and mTORC1 signaling during SD. (**A**) Representative western blots of acute (15 min to 8 hr) AMPKα signaling. (**B**–**D**) Western blot quantification of acute AMPKα related signaling events. (**E**) Representative western blots of chronic (2–4 days) AMPKα signaling. (**F**–**H**) Western blot quantification of chronic AMPKα related signaling events. (**I**) Representative western blots of acute mTORC1 signaling. (**J**–**L**) Western blot quantification of acute mTORC1 related signaling events. (**M**) Representative western blots of chronic mTORC1 signaling. (**N**–**P**) Western blot quantification of chronic mTORC1 related signaling events. $^{****}p < 0.0001$, $^{***}p < 0.001$, $^{**}p < 0.01$, $^{*}p < 0.05$. N = 4–6/group. Abbreviations: ACC: Acetyl-CoA carboxylase; AMPKα: 5’ AMP-activated protein kinase alpha; PGC-1α: PPARG coactivator 1alpha; GAPDH: Glyceraldehyde 3-phosphate dehydrogenase; mTORC1: Mechanistic target of rapamycin complex 1; p70S6K: Ribosomal protein S6 kinase; rpS6: Ribosomal protein S6.

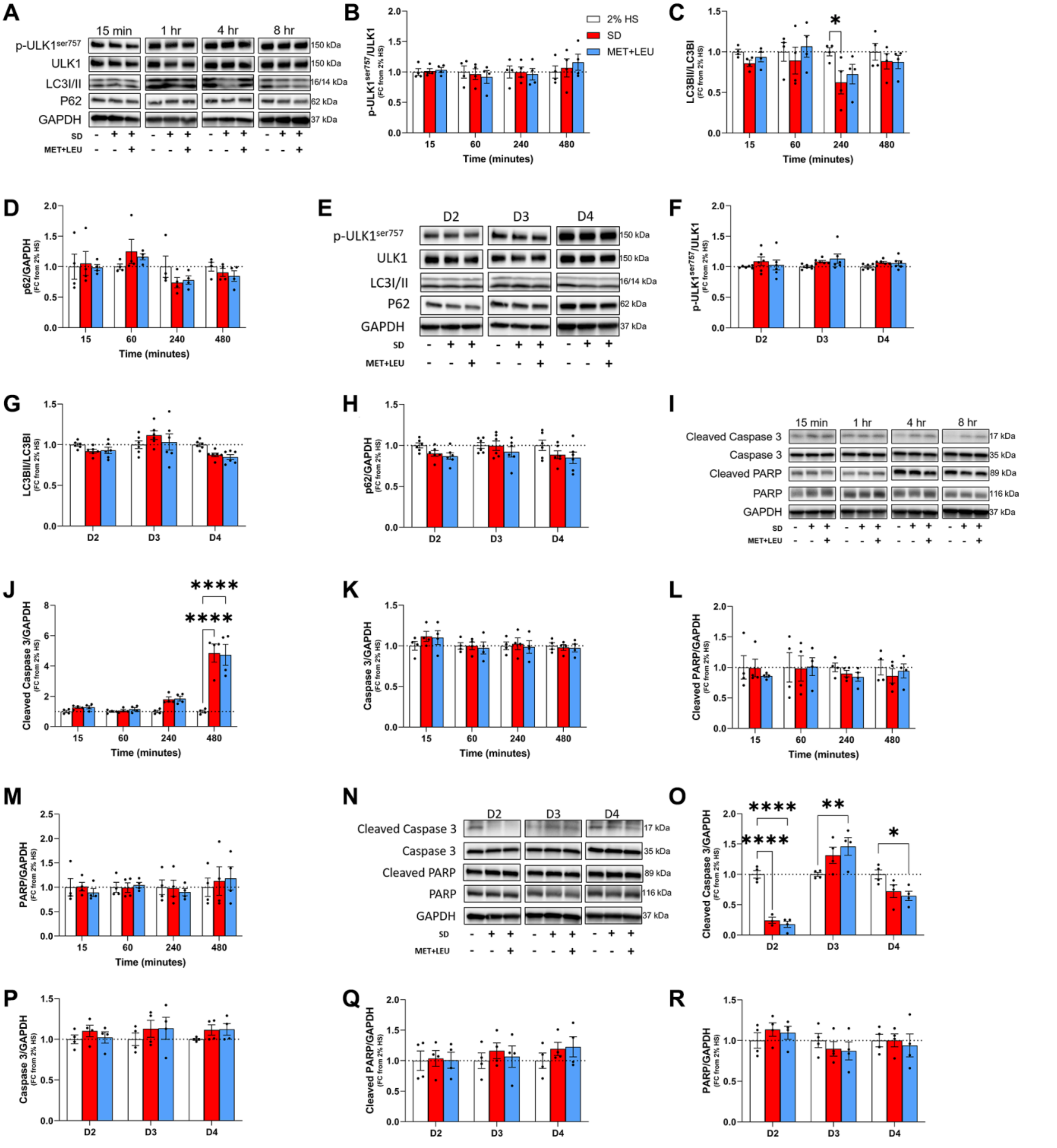

Supplementary Figure 6. Acute and chronic autophagy and apoptosis markers during SD. (**A**) Representative western blots of autophagy markers following 15 min to 8 hr of SD and treatments. (**B**–**D**) Western blot quantification of autophagy markers at acute treatment timepoints. (**E**) Representative western blots of autophagy markers following 2 to 4 days of SD and treatments. (**F**–**H**) Western blot quantification of apoptosis markers following 2 to 4 days of SD and treatments. (**I**) Representative western blots of apoptosis markers following 15 min to 8 hr of SD and treatments. (**J**–**M**) Western blot quantification of apoptosis markers at acute treatment timepoints. (**N**) Representative western blots of apoptosis markers following 2 to 4 days of SD and treatments. (**O**–**R**) Western blot quantification of apoptosis markers at chronic treatment timepoints. $^{****}p < 0.0001$, $^{**}p < 0.01$, $^{*}p < 0.05$. N = 4–6/group. Abbreviations: ULK1: Unc-like autophagy activating kinase 1; LC3I/II: Microtubule-associated protein light chain 3–1/2; P62: Sequestosome 1; GAPDH: Glyceraldehyde 3-phosphate dehydrogenase; PARP: Poly (ADP-ribose) polymerase.

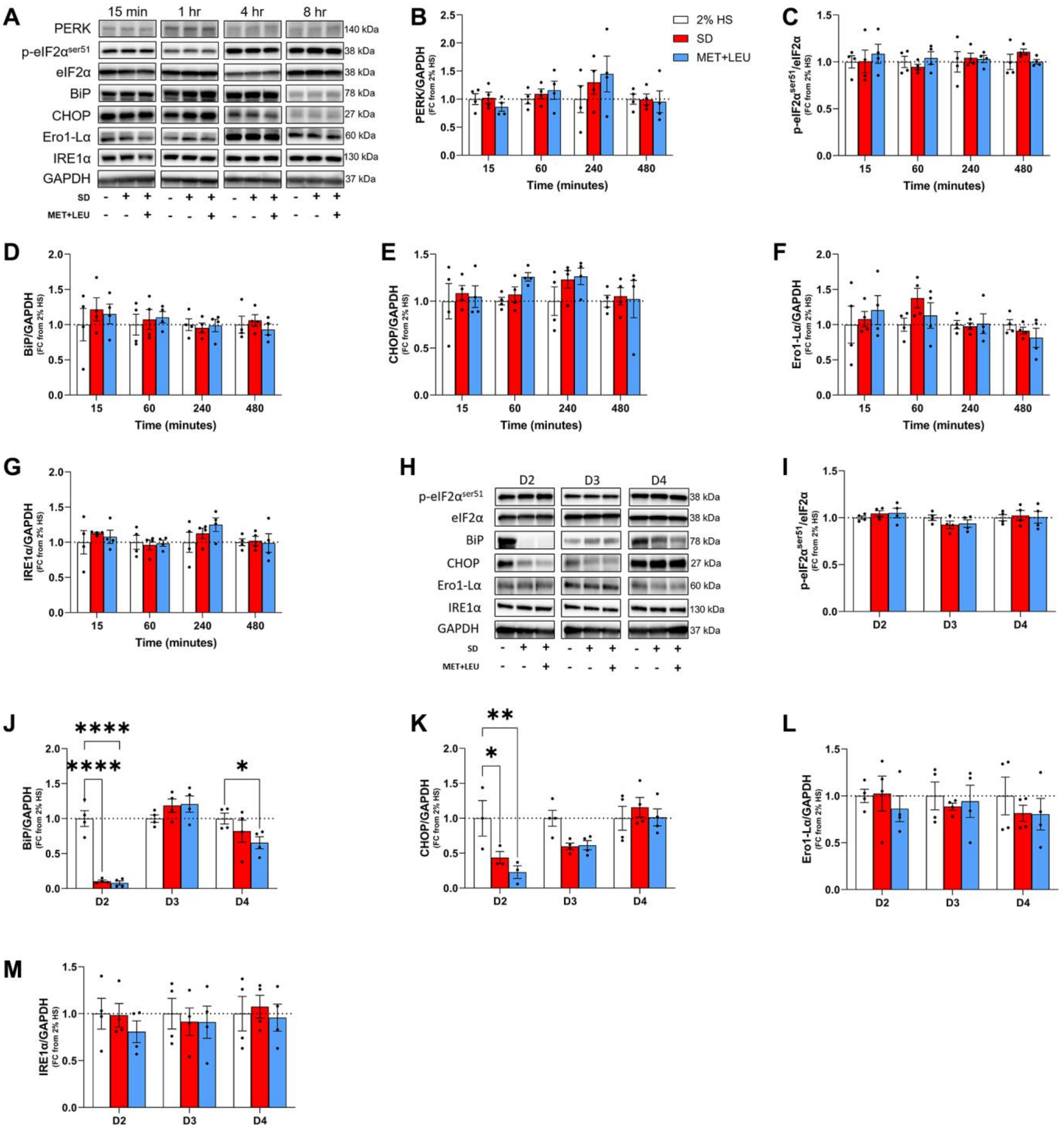

Supplementary Figure 7. ER stress markers during SD. (**A**) Representative western blots of ER stress markers following 15 min to 8 hr of SD. (**B**–**G**) Western blot quantification of ER stress markers at acute treatment timepoints. (**H**) Representative western blots of ER stress markers following 2 to 4 days of SD and treatments. (**I**–**M**) Western blot quantification of ER stress markers at chronic treatment timepoints. $^{****}p < 0.0001$, $^{**}p < 0.01$, $^{*}p < 0.05$. $N = 4$/group. Abbreviations: PERK: Protein kinase RNA-like ER kinase; eIF2α: Eukaryotic translation initiation factor 2 alpha; BiP: Binding immunoglobin protein; CHOP: C/EBP homologous protein; Ero1-Lα: Endoplasmic reticulum oxidoreductase 1 alpha; IRE1α: Inositol-requiring enzyme 1 alpha; GAPDH; Glyceraldehyde 3-phosphate dehydrogenase.

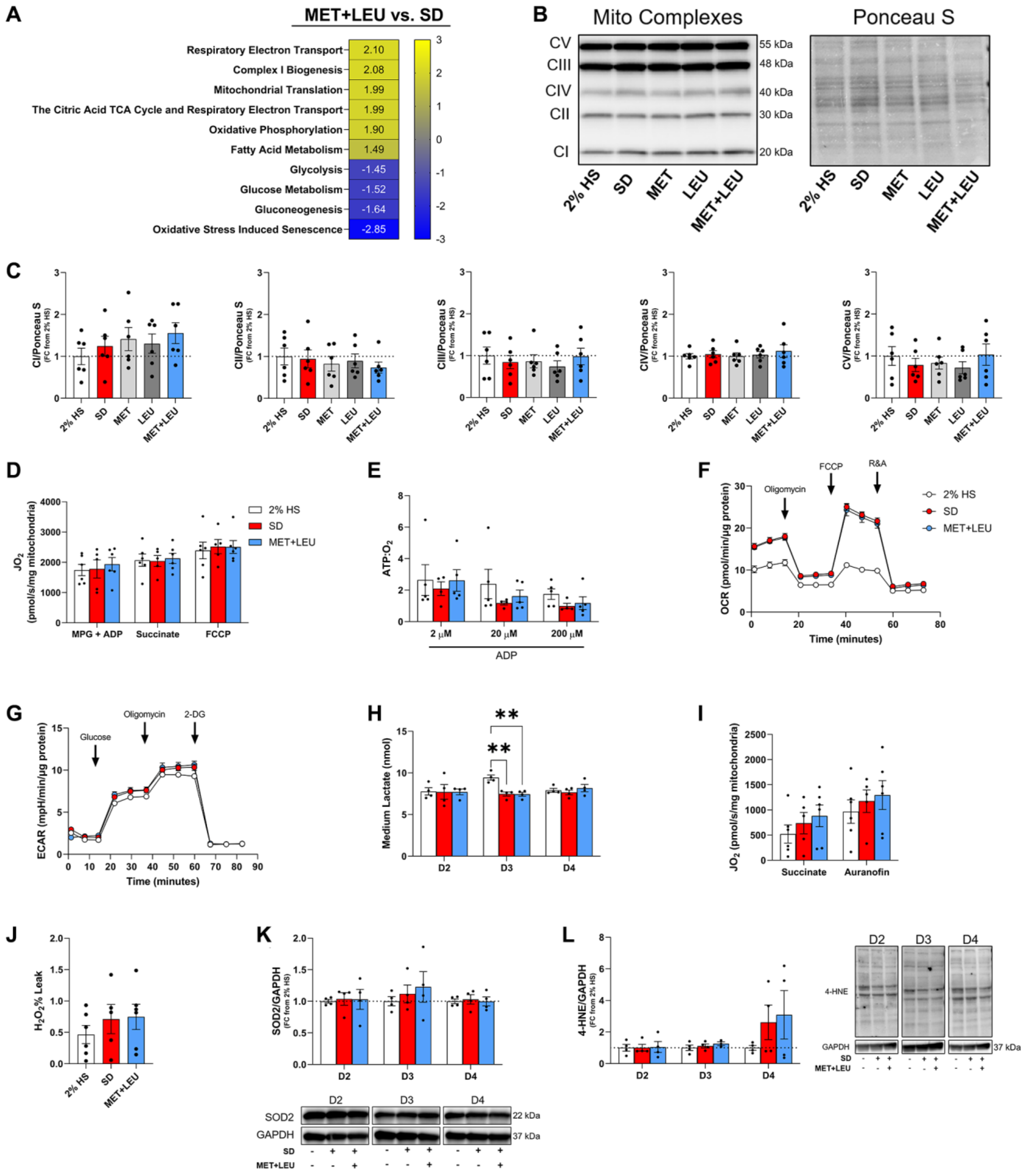

Supplementary Figure 8. MET+LEU improved transcriptional pathways compared to SD without functional changes in metabolism. (**A**) After 4-days of SD, transcriptional pathways related to mitochondrial content and function in MET+LEU vs. SD represented as NES. (**B**) Representative western blots of mitochondrial complex proteins. (**C**) Western blot quantification of mitochondrial complex proteins. (**D**) In isolated mitochondria, mitochondrial respiration with substrates malate, pyruvate, glutamate (MPG) + adenosine diphosphate (ADP), succinate, then carbonyl cyanide p-trifluoro-methoxyphenyl (FCCP). (**E**) In isolated mitochondria, mitochondrial efficiency measured by the ratio of ATP production: oxygen consumption. (**F**) Whole cell oxygen consumption rate (OCR). (**G**) Whole cell glycolytic function measured via extracellular acidification rate (ECAR). (**H**) Lactate in media from myotubes following 2, 3, or 4-days of SD. (**I**) ROS production (succinate) and emission (auranofin). (**J**) Hydrogen peroxide leak percentage. (**K**) Superoxide dismutase 2 (SOD2) western blot quantification and representative image. (**L**) Lipid peroxidation measured via 4-Hydroxynonenal (4-HNE), representative western blot image and quantification. $^{**}p < 0.01$, N = 4–6/group.

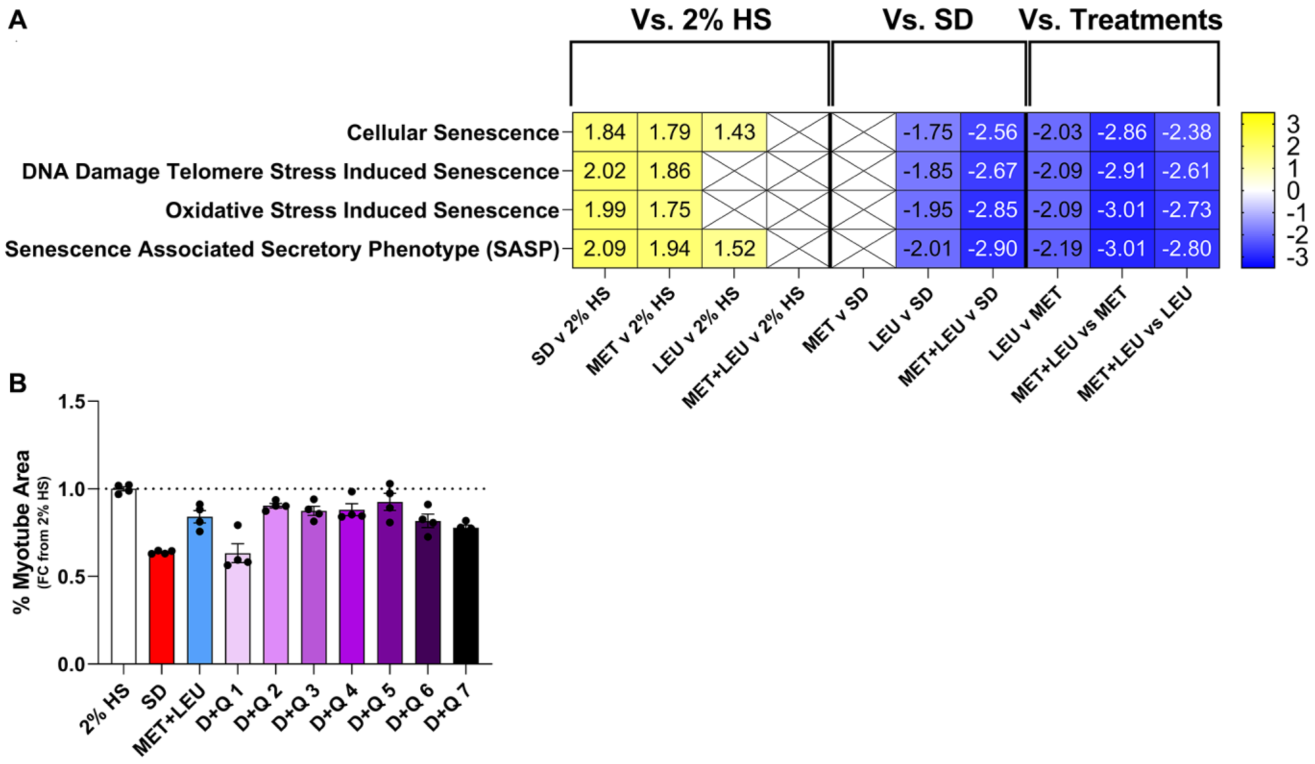

Supplementary Figure 9. MET+LEU effect on cellular senescence pathways is likely driven by LEU synergy with MET. (**A**) Pathways altered in all groups vs. 2% HS, SD, and the treatments compared to each other, related to cellular senescence; numbers are representative of NES. (**B**) Dose-response experiment of dasatinib + quercetin (D+Q); doses are the following: (1) 125 nM D + 25 µM Q, (2) 62.5 nM D + 12.5 µM Q, (3) 31.25 nM D+ 6.25 µM Q, (4) 15.63 nM D + 3.13 µM Q, (5) 7.81 nM D + 1.56 µM Q, (6) 3.91 nM D + 781.25 nM Q, (7) 1.95 nM D + 390.63 nM Q. *N* = 6/group for (**A**), *N* = 4/group for (**B**).

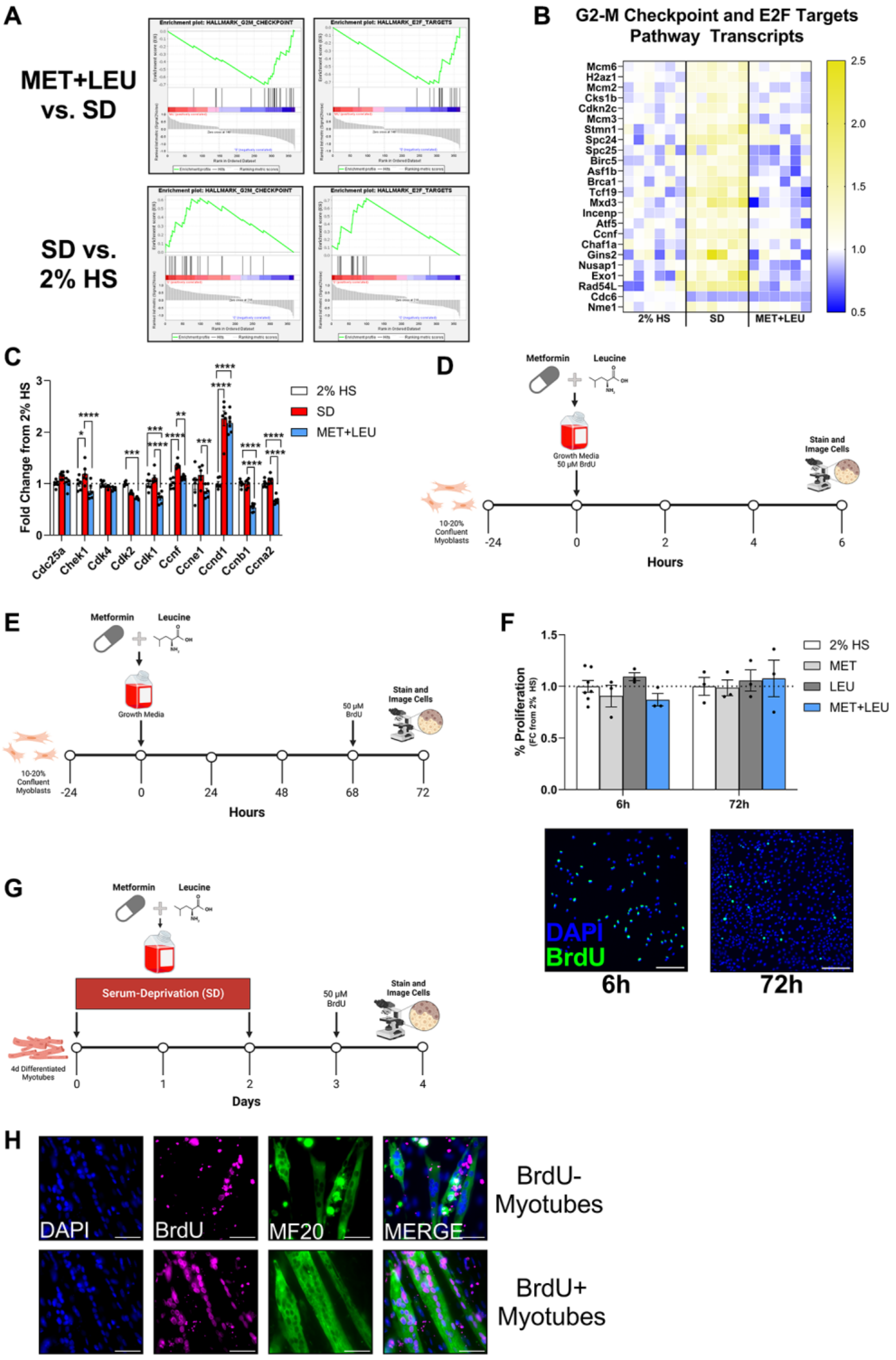

Supplementary Figure 10. Cell cycle pathways, myoblast and myotube nuclei proliferation during SD. (**A**) After 4-days SD in the 378 MET+LEU reversed genes, G2M checkpoint and E2F target gene set enrichment analysis (GSEA) pathways with MET+LEU vs. SD and SD vs. 2% HS. (**B**) Heatmap of G2M checkpoint and E2F target gene changes as fold change from 2% HS. (**C**) Cell cycle related transcripts from RNA-sequencing dataset. (**D**) Experimental schematic of 6-hour treatment with BrdU labeling on myoblast proliferation. (**E**) Experimental schematic of 72-hour treatment with 4-hour BrdU labeling on myoblast proliferation. (**F**) Percent myoblast proliferation after 6 or 72-hour treatments, with representative images, scale bar = 200 µm. (**G**) Experimental schematic used to determine BrdU incorporation into myotubes following 4-days SD. (**H**) Representative image of BrdU incorporation into myotubes. *N* = 3–6/group.
